# Pseudoaneurysm of the aortic arch: A rare case report of pulmonary cancer complication

**DOI:** 10.1097/MD.0000000000004457

**Published:** 2016-08-07

**Authors:** Yuan-Qiang Lu, Feng Yao, An-Dong Shang, Jian Pan

**Affiliations:** Department of Emergency Medicine, First Affiliated Hospital, School of Medicine, Zhejiang University, Hangzhou, People's Republic of China.

**Keywords:** aortic arch pseudoaneurysm, computed tomographic angiography, endovascular stent-grafting, hemoptysis, pulmonary cancer

## Abstract

**Background:**

Pseudoaneurysm of the aortic arch is uncommonly associated with cancer, and is extremely rare in pulmonary cancer. Here, we report an unusual and successfully treated case of aortic arch pseudoaneurysm in a male patient with lung squamous cell carcinoma.

**Methods:**

A 64-year-old male patient was admitted to the Emergency Department, presenting with massive hemoptysis (>500 mL blood during the 12 hours prior to treatment). The diagnosis of aortic arch pseudoaneurysm was confirmed after inspection of computed tomographic angiography and three-dimensional reconstruction. We processed the immediate endovascular stent-grafting for this patient.

**Results:**

This patient recovered with no filling or enlargement of the pseudoaneurysm, no episodes of hemoptysis, and no neurological complications during the 4-week follow-up period.

**Conclusion:**

Herein, we compare our case with other cancer-related pseudoaneurysms in the medical literature and summarize the clinical features and treatment of this unusual case.

## 1 Introduction

Pseudoaneurysms result from a variety of causes, including trauma, infection, vasculitis, and artherosclerosis or iatrogenicities.^[1]^ As a relatively fatal disease, aortic pseudoaneurysm has a high mortality rate as a result of the high tension of the edematous aortic wall, which causes the rupture.^[2,3]^ Therefore, this disease should be treated soon after diagnosis. Currently, there are no reports about spontaneous aortic arch pseudoaneurysm rupture causing hemoptysis in patients with pulmonary cancer. In this study, the endovascular stent-grafting treatment provided a good outcome for the pulmonary cancer patient, who presented with spontaneous aortic arch pseudoaneurysm bleeding.

## 2 Case presentation

A 64-year-old Chinese male patient was admitted to the Emergency Department, presenting with massive hemoptysis (>500 mL blood during the 12 hours prior to treatment), accompanied by chest distress and shortness of breath. The patient was a tobacco smoker (one pack of cigarettes daily for 40 years) and had taken amlodipine besylate to treat hypertension for 7 years. No history of previous cardiovascular or respiratory diseases or trauma was recorded. The patient had a temperature of 37.4°C, heart rate of 123 beats/min, respiratory rate of 35 breaths/min, and blood pressure of 127/82 mm Hg (1 mm Hg = 0.133 kPa). The general physical examination was unremarkable, except for rough sound of breath heard on the pulmonary examination.

**Figure 1:**
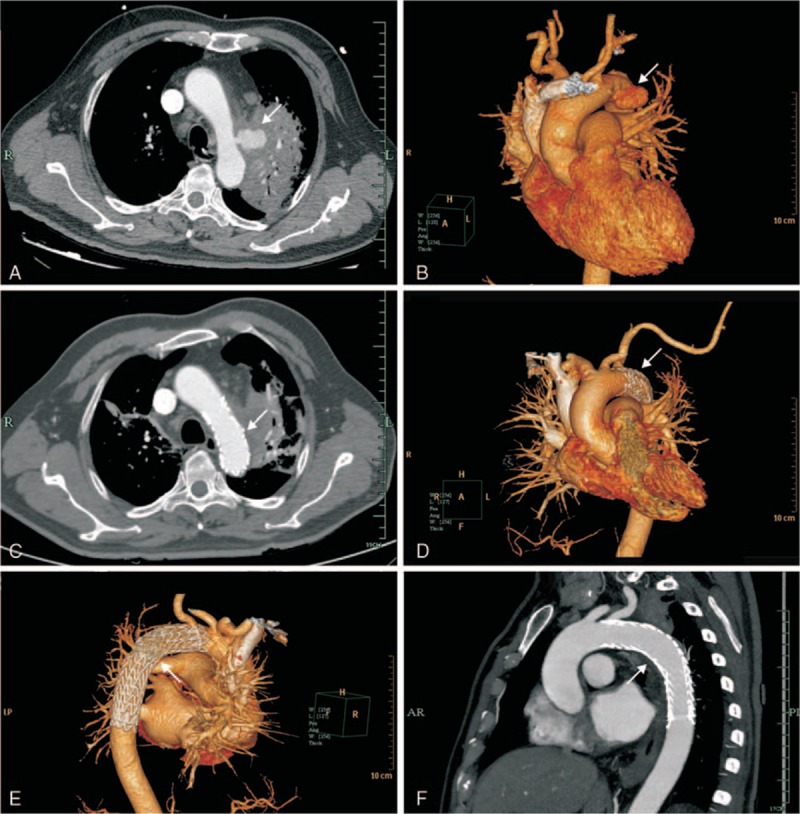
Preoperative CTA: thoracic CT scan showing an irregular wall at the level of the aortic arch, which is an aortic arch pseudoaneurysm (A, B). Follow-up CTA: CTA on the 5th postoperative day showed the shape and the location of the stent-graft were satisfactory, and no evidence of endoleak, filling, or enlargement of the pseudoaneurysm (C–F). CT = computed tomography, CTA = computed tomography angiography.

On admission, the laboratory characteristic results for this patient were as shown in Table 1, which indicated infection, mild metabolic acidosis, and impaired cardiac function. The liver and renal function tests were all normal. Sinus tachycardia was found on the electrocardiogram, which was consistent with the characteristics of hemorrhage. Consequently, pulmonary enhanced computed tomography (CT) detected a space-occupying lesion in the left upper lobe of the lung with peripheral obstructive pneumonia, as well as a suspected aortic arch pseudoaneurysm. The images of further computed tomographic angiography (CTA) and three-dimensional (3D) reconstruction were consistent with the diagnosis shown on the pulmonary enhanced CT. CTA images revealed bilateral lung infection, the leakage of the pseudoaneurysm at the distal part of the left subclavian artery (LSA), and the space-occupying lesion in the left upper lung, which just encircled the pseudoaneurysm (Fig. 1A and B). Multiple mediastinal lymph node enlargements were also found. Taking into account the bleeding of the aortic arch pseudoaneurysm, the endovascular stent graft (31 mm × 150 mm, W.L. Gore & Associates, Flagstaff, AZ) was promptly implanted in this patient, under general endotracheal anesthesia and digital subtraction angiography. The operation went smoothly, and postoperative angiography showed that the shape and location of the stent-graft were satisfactory; with no endoleak or migration observed. After surgery, this patient was transferred to the Emergency Intensive Care Unit (EICU) for continuous monitoring. Antibiotics, hemostatic medications, and fluids were continuously administered intravenously for 3 days.

**Table 1:**
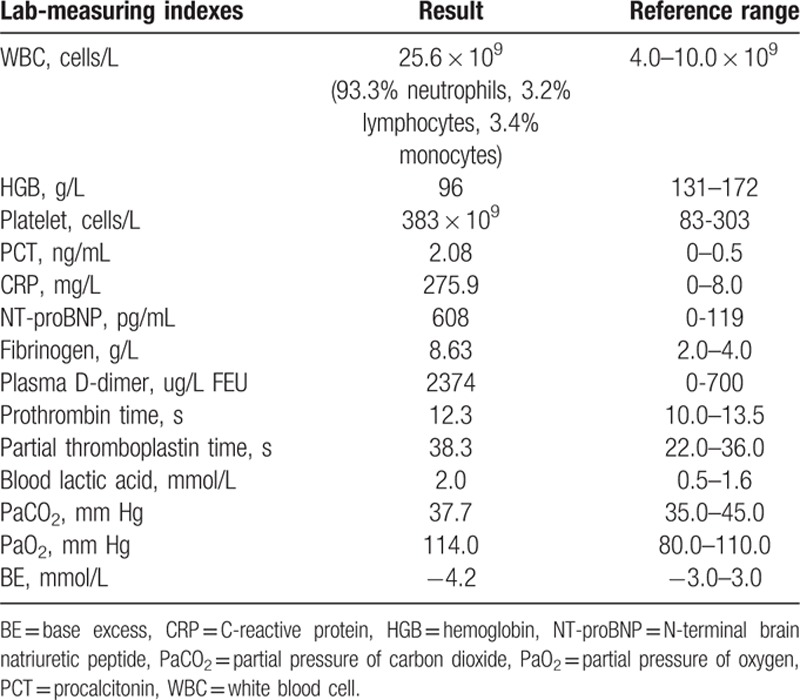
The characteristic results of the patient underwent the laboratory investigations on admission.

The patient recovered favorably from the operation, without complications. The severe hemoptysis was controlled immediately after surgery, except for a little blood-tinged sputum. The above symptoms, including the chest pain and shortness of breath, disappeared on the 4th postoperative day. On the 5th day, follow-up CTA showed no filling or enlargement of the pseudoaneurysm (Fig. 1C–F). Meanwhile, the space-occupying lesion in the left upper lobe of the lung was considered to be malignant, based on a positron emission tomography (PET)-CT examination after the operation. Later, a bronchoscopic biopsy revealed squamous cell carcinoma of the lung. On the 10th day, the procalcitonin (PCT) level decreased to 0.05 ng/mL (reference range 0–0.5 ng/mL) and the C-reactive protein (CRP) level dropped to 42.10 mg/L (reference range 0–8.0 mg/L). However, the related biochemical indicators, including blood lactic acid, arterial blood gas, and serum N-terminal brain natriuretic peptide (NT-proBNP), returned to normal. He was discharged uneventfully from the hospital after 14 days, and his white blood cell count (WBC) decreased to 7.6 × 10^9^ cells/L with 65.1% neutrophils. No recurrent episodes of hemoptysis or neurological complications were noted in the following 4 weeks. The patient was transferred to the Department of Oncology for further treatment of pulmonary cancer after 2 weeks of recovery.

Written informed consent was obtained from the patient when he was admitted to our hospital. The study was approved by the Research Ethics Committee of First Affiliated Hospital, College of Medicine, Zhejiang University. We performed a search on PubMed (National Library of Medicine, Bethesda, MD) using the terms “Pseudoaneurysm” and “Pulmonary cancer” retrieving all available articles published in English up to March 2016. We combined these data with our new case report to describe the characteristics of this aortic arch pseudoaneurysm case.

## 3 Discussion

According to the above clinical data, the lung squamous cell carcinoma complicated by aortic arch pseudoaneurysm mainly caused the massive hemoptysis in this patient. It is well known that pulmonary cancer is one of the common causes of hemoptysis; sometimes it even causes massive bleeding.^[4]^ The massive hemoptysis, which is considered a life-threatening condition, requires effective and timely management.^[5,6]^ In our case, since this patient's hemoptysis was controlled rapidly after the endovascular stent graft implantation, we believe that the massive hemoptysis in this patient resulted from the leaking or bleeding of the complicated aortic arch pseudoaneurysm, rather than the pulmonary cancer alone.

In addition, the results of the initial hemogram showed the high WBC count with an elevated neutrophil count, which we speculated were as a result of the infection, stress, or leukemoid reaction.^[7]^ However, the WBC count returned to normal after endovascular stent graft placement and antibiotic treatment; therefore, the possibility of leukemoid reaction was primarily ruled out.

Pseudoaneurysm of the aortic arch is usually a rare complication after thoracic surgery or trauma; it is fatal when the rupture occurs from the power of the aortic flow. Moreover, aortic arch pseudoaneurysm due to cancer is generally uncommon and is extremely rare in pulmonary cancer. There are a few reports on cancer-related pulmonary vasculature pseudoaneurysms, and some described cases of pulmonary vasculature pseudoaneurysms arising in aggressive lung squamous cell carcinoma.^[8–11]^ Small pulmonary vasculature pseudoaneurysm lesions under low pressure often resolve spontaneously when the mean pulmonary artery pressure (MPAP) is 12 to 16 mm Hg. However, the mean artery pressure (MAP) of healthy people is 70 to 105 mm Hg, approximately 6 times MPAP. In our case, this patient's MAP was 78 mm Hg on initial physical examination in our hospital, and this cancer-related pseudoaneurysm growth after aggressive lung squamous cell carcinoma was a result of vascular destruction. In addition, hypertension is a well-known cause of vascular degeneration and atherosclerosis.^[12]^ Combined with the pulmonary tumor invasion, the prolonged hypertension of this patient might cause the weakening of the aortic wall, and increase the incidence of pseudoaneurysm formation. Thus, this aortic arch pseudoaneurysm lesion under high pressure requires urgent intervention to prevent life-threatening hemoptysis.

The pseudoaneurysm is associated with a high mortality rate of 61% with nonsurgical treatment, owing to documented rupture.^[13]^ Conventional open surgery for pseudoaneurysm remains a surgical challenge because it is associated with a high rate of mortality (7–17%) and neurological complications (4–12%).^[14–17]^ In this case, we applied endovascular stent-grafting, which was a minimally invasive treatment, for thoracic aortic disease. Endovascular procedures have been more commonly used for the treatment of aortic arch pseudoaneurysm recently.^[2,14,18,19]^ Compared with conventional open surgery, endovascular stent-grafting is less invasive, without any dissection, and having less bleeding and a shorter procedural time and length of hospital stay. However, long-term clinical efficacy and safety of this operation have not been confirmed yet.^[2,14,15]^ Therefore, the long-term outcome of this procedure needs to be observed in future research.

Pseudoaneurysm can present as hemoptysis, chest pain, and shortness of breath.^[8]^ In this case, hemoptysis was caused by bleeding of the aortic arch pseudoaneurysm, which results from erosion by pulmonary cancer. It is worth noting that the patient maintained stable vital signs from the start of the hemoptysis to the operation, during which time he was bleeding from the arterial pseudoaneurysm for more than 15 hours. This may be due to a relative slow rate of pseudoaneurysm leaking or bleeding effectively hindered by the pulmonary cancer tissue near the rupture. This dense tissue adhesion possibly results from intercellular adhesion molecule-1 (ICAM-1) expression from invasive lung tumors.^[20]^ Unfortunately, our case with end-stage cancer did not meet the indications for tumor resection; thus, we could not get the large tumor sample to analyze and verify the expression of these intercellular adhesion molecules. This still needs to be elucidated with further research.

To our knowledge, this is a rare report of an aortic arch pseudoaneurysm as a complication of pulmonary cancer. Although the detailed mechanism causing aortic arch pseudoaneurysm from pulmonary squamous cell carcinoma remains unclear, and the prognosis value after successful endovascular stent-grafting still needs to be evaluated under long-term monitoring, aortic arch pseudoaneurysm could be recognized as a severe complication of pulmonary tumors adjacent to the aortic artery.
